# Rapid assessment of the performance of malaria control strategies implemented by countries in the Amazon subregion using adequacy criteria: case study

**DOI:** 10.1186/1475-2875-10-379

**Published:** 2011-12-20

**Authors:** Walter Flores, Jaime Chang, Edgar Barillas

**Affiliations:** 1 Strengthening Pharmaceutical Systems/Management Sciences for Health, 6a avenida 11-77 zona 10, Edificio Punto Diez oficina 1 F Guatemala City, Guatemala; 2 Office of Health USAID Peru, Av. La Encalada Cuadra 17 s/n Santiago de Surco, Lima 33, Peru; 3 Strengthening Pharmaceutical Systems/Management Sciences for Health, Suite 400, 4301 North Fairfax Drive, Arlington VA 22203-1627, USA

## Abstract

**Background:**

The objective of this study was to implement a rapid assessment of the performance of four malaria control strategies (indoor spraying, insecticide-treated bed nets, timely diagnosis, and artemisinin-based combination therapy) using adequacy criteria. The assessment was carried out in five countries of the Amazon subregion (Bolivia, Colombia, Ecuador, Guyana, and Peru).

**Methods:**

A list of criteria in three areas was created for each of the four strategies: preliminary research that supports the design and adaptation of the control strategies, coverage of the control strategies and quality of the implementation of the strategies. The criteria were selected by the research team and based on the technical guidelines established by the World Health Organization. Each criterion included in the four lists was graded relative to whether evidence exists that the criterion is satisfied (value 1), not satisfied (value 0) or partially satisfied (value 0.5). The values obtained were added and reported according to a scale of three implementation categories: adequate, intermediate and deficient.

**Results:**

Implementation of residual indoor spraying and timely diagnosis was adequate in one country and intermediate or deficient in the rest. Insecticide-treated bed nets ranged between deficient and intermediate in all the countries, while implementation of artemisinin-based combination therapy (ACT) was adequate in three countries and intermediate in the other two countries evaluated.

**Conclusions:**

Although ACT is the strategy with the better implementation in all countries, major gaps exist in implementation of the other three malaria control strategies in terms of technical criteria, coverage and quality desiredThe countries must implement action plans to close the gaps in the various criteria and thereby improve the performance of the interventions. The assessment tools developed, based on adequacy criteria, are considered useful for a rapid assessment by malaria control authorities in the different countries.

## Background

In the last decade, the epidemiology of malaria has changed quite significantly in the Americas. Between 2000 and 2009, the number of cases reported for the region decreased by 50%, from 1.18 million to 526,000, while the variation within the countries included a 90% decrease in Suriname and almost a 200% increase in Haiti. The ratio between the number of cases of *Plasmodium vivax *malaria and *Plasmodium falciparum *malaria did not vary greatly, holding steady at approximately 3:1 [1].

The Amazon subregion continues to report about 90% of the total malaria cases for the Americas, as well as the majority of *P. falciparum *cases, although the absolute number of cases in 2000-2008 decreased from 992,061 to 515,440, or 50% [2], similar to the regional trend.

Evaluating the impact of the different control strategies on the reduction of malaria described above is an important task; however, it is an extremely complex task with high costs. In addition, the positive changes described probably do not result solely from the control strategies, but are also the result of a combination of factors that include changes in the environmental and epidemiological conditions determining the transmission of malaria.

Because public health interventions are complex, it is important to be clear about the specific aspects, programmes, and interventions to be evaluated. Habicht et al. [3] propose a logical framework to evaluate the performance and impact of public health interventions. This framework has been used in various international studies [4-7]. The design comprises three levels, ranging from simple to complex, depending on the evaluation designs and type of inference one wishes to make: (a) adequacy, (b) plausibility and, (c) probability. The adequacy level is the most basic and refers to evaluation of public health interventions relative to criteria generally corresponding to technical aspects of the interventions, quality of implementation and coverage of services. The inference that is made in an adequacy evaluation is whether the interventions are being implemented as planned and achieving the desired objectives (i.e., coverage, quality or others). The plausibility level requires a control group, and the inference indicates the possibility that the effects observed in the intervention group result from the intervention and not external factors. The probability level requires that the control group be assigned randomly, and the inference is presented in terms of effects in the intervention group being results of the intervention with known certainty and statistical reliability, and not results of mixed variables, bias or chance.

The Habicht et al. [3] framework is also useful in identifying the various levels of evidence required to make decisions about a public health intervention. For example, if one has no knowledge or evidence that an intervention is being implemented adequately, according to technical standards and with the necessary resources, then a probabilistic evaluation cannot be considered because any effects and impact the evaluation finds cannot be attributed to the intervention. In addition, if the intervention has not been implemented in an adequate manner, one can anticipate that it will have no impact. In these cases, one must first evaluate the adequacy of the interventions. Application of the logic described above also contributes to avoiding unnecessary expenses in evaluation studies when simpler evaluations can be carried out [7].

Based on the framework proposed by Habitch et al. [3], the adequacy level was chosen for this study which involved evaluating whether the malaria control strategies are implemented in a technically correct manner, with the necessary resources and required quality.

The following malaria control strategies were evaluated: (a) indoor residual spraying, (b) insecticide-treated bed nets, (c) timely diagnosis and, (d) artemisinin-based combination therapy (ACT). The five countries studied were Bolivia, Colombia, Ecuador, Guyana and Peru.

## Methods

From the beginning, the intention of the research team was to carry-out a rapid assessment using secondary data and applying the adequacy level of the Habitch et al. framework [3]. A literature search in *PubMed *was first carried out using key words to identify experiences with evaluation of malaria control strategies, rapid assessments and evaluation of public health interventions using the Habitch et al. framework [3]. A second part of the literature search involved identifying and collecting the technical guidelines for malaria control strategies produced by the World Health Organization (WHO). Those guidelines were identified through the WHO's web site search engine.

Although there are rapid assessments approaches that have been implemented in the region and elsewhere [8-10] they either rely on the collection of primary data or do not apply the adequacy framework [3].

The next step was to develop the framework that were to be applied in this study For this,a list of criteria was established for each of the four strategies in three thematic areas: (a) preliminary research supporting the design and adaptation of the control strategies, (b) coverage of the control strategies and, (c) quality of the implementation of the strategies. The criteria were selected by the research team based on the technical guidelines established by the World Health Organization (WHO) [11-14].

Each criterion included in the four lists was graded relative to whether evidence exists that the criterion is satisfied (value 1), not satisfied (value 0), or partially satisfied (value 0.5). The values obtained were added and reported according to a scale with three categories: adequate, intermediate and deficient implementation. The cut-off point for each of the three categories was decided by the researchers following practical assumptions: a) although in the strict sense adequate implementation should require all criteria to be fulfilled, it was important to introduce some flexibility to motivate national programmes towards improving performance, hence a flexible understanding of adequacy was introduced to represent 80% and higher of critera fulfilled; b) Anything equivalent to 50% or lower of criteria fulfilled should be classified as deficient and c) all values between 51% and 79% should be considered as intermediate adequacy. To ease understanding of the scale, each category was converted to cardinal numbers. Tables 1, 2, 3, 4 present the grading scale and the criteria for each one of the four malaria control strategies.

**Table 1:** Adequacy criteria for implementation of indoor residual spraying

	GRADE: Total criteria: 9 7.5 to 9 criteria satisfied = Programme implementation adequate 5 to 7 criteria satisfied = Programme implementation of intermediate adequacy 0 to 4.5 criteria satisfied = Programme implementation deficient
**No.**	**Criteria**

	***Preliminary research phase***

1	The population at risk was stratified according to the burden of the disease and epidemiology of the transmission [10]

2	Vector habits were analysed and confirmed [10]

3	Sensitivity to potential insecticides was confirmed prior to selecting the insecticide(s) with the best results [10]

	***Coverage***

4	100% of the target houses (according to national standards) were sprayed at least once a year [9]

5	Stockouts of insecticides for spraying did not exceed six months in any of the cases [9]

	***Quality***

6	Updated programmes and rules exist on the implementation of residual spraying [10]

7	A system is in place to monitor the resistance and sensitivity of insecticides used in indoor spraying [10]

8	Systematic procedures exist to monitor vector habits [10]

9	Systematic procedures exist to monitor residual insecticide in the homes sprayed [10]

**Table 2:** Adequacy criteria for insecticide-treated bed net strategy

	GRADE: Total criteria: 14 11.5 to 14 criteria satisfied = Programme implementation adequate 7.5 to 11 criteria satisfied = Programme implementation of intermediate adequacy 0 to 7 criteria satisfied = Programme implementation deficient
**No.**	**Criteria**

	***Preliminary research phase***

1	The population at risk was stratified according to the burden of the disease and epidemiology of the transmission [8]

2	Vector habits were analysed and confirmed [8]

3	Sensitivity to potential insecticides was confirmed before selecting the insecticide(s) with the best results [8]

	***Coverage***

4	80% of the population at risk has received insecticide-treated bed nets [7]

5	Insecticide-treated bed nets were distributed to 80% of pregnant women in the risk area [7]

6	Insecticide-treated bed nets were distributed to 80% of children under age five in the risk areas [7]

7	80% of those surveyed said that they had slept under a bed net the night before [7]

8	Stockouts of insecticide for impregnation [of bednets] did not exceed three months in the last five years [7]

9	Stockouts of new bed nets for delivery to the population did not exceed six months in any of the cases [7]

	***Quality***

10	Standards and programmes exist for reimpregnation [of bed nets] on the household or community level [8]

11	A systematic procedure exists for monitoring that families have bed nets and use them appropriately (including re-impregnation and washing) [8]

12	A system is in place to monitor the resistance and sensitivity of insecticides used in bed nets [8]

13	Systematic procedures exist to monitor vector habits [8]

14	Systematic procedures exist to monitor residual insecticide in the bed nets [8]

**Table 3:** Adequacy criteria for timely diagnosis

	GRADE: Total criteria: 7 6 to 7 criteria satisfied = Programme implementation adequate 4 to 5.5 criteria satisfied = Programme implementation of intermediate adequacy 0 to 3.5 criteria satisfied = Programme implementation deficient
**No.**	**Criteria**

	***Coverage***

1	At least 80% of all cases are diagnosed in the first 24 hours (time between taking the thick-smear microscopy or rapid diagnostic test and delivery of the results in endemic areas) [7]

2	There were no stockouts of rapid diagnostic tests in any of the public network facilities in the endemic areas [7]

	***Quality***

3	A system exists for monitoring the quality of microscopic diagnosis in the public network [9]

4	A system exists for monitoring the quality of rapid diagnostic tests [10]

5	There are national standards for the use, distribution, shipment and storage of rapid diagnostic tests [10]

6	A systematic process exists for monitoring compliance with distribution, shipment and storage standards [10]

7	Some type of training and supervision programme exists for the staff that uses rapid diagnostic tests [10]

**Table 4:** Adequacy criteria for the ACT strategy

	GRADE: Total criteria: 8 6.5 to 8 criteria satisfied = Programme implementation adequate 4.5 to 6 criteria satisfied = Programme implementation of intermediate adequacy 0 to 4 criteria satisfied = Programme implementation deficient
**No.**	**Criteria**

	***Preliminary studies***

1	*In vivo *or *in vitro *studies on the resistance of *P. falciparum *[to] medications in previous treatment plans were carried out [11]

2	*In vivo *or *in vitro *studies on the sensitivity of *P. falciparum *to ACT were carried out [11]

	***Coverage***

3	At least 80% of *P. falciparum *cases receive ACT [7]

4	No ACT stockouts occurred in the public network in the last three years [7]

	***Quality***

5	Updated protocols and standards exist for ACT in the country's treatment plans [11]

6	A system is in place for monitoring ACT treatment failures [11]

7	According to standards, ACT is only given when a positive result (microscopic or rapid diagnostic test) occurs for *P. falciparum*

8	A systematic procedure exists to monitor the appropriate application of standards and protocols for ACT [11]

For data collection, several tools were designed. First, detailed and comprehensive questionnaires addressing each one of the criterium for the four interventions were developed. These questionnaires were sent by email, with a request to completion, to the national directors of malaria control programmes in each country. Due to the nature of the questions, each country involved a team of national experts to respond the questions and provide the required means of verification. A visit to each country by field assistants followed to have face-to-face meetings with national authorites to clarify returned information and verify responses.

A second set of data collections tools were interview guidelines to the following actors: a) national malaria authorities b) procurement directors and c) malaria technical advisors from the Pan American Health Organization in each country and at the regional level. These guidelines aimed to expand issues identified in the first questionnaires (i.e. problems with procurement, absence of technical studies before introducing interventions, challenges imposed by ongoing health reforms) and to collect interviewees' perceptions regarding the preliminary scoring of adequacy based on the responses to the first questionnaires. Interviews to all the actors above in each country were carried-out face-to-face by the principal researchers. The questionnaires and interview guidelines are available as a supplement.

For triangulation purposes, every response to the questionnaires by malaria national programmes had to be accompanied by specific means of verification (i.e. treatment guidelines, copies of the reports of the specific studies on resistance to antimalarials, vector habits) For coverage, information analysed related to the period 1995-2008. Coverage indicators provided by national authorities were contrasted with the information compiled in the electronic databases available at the Pan American Health Organization's website. In the few cases in which discrepancy was observed, the information provided by national authorities prevailed.

The principal researchers analysed all collected data. The interview to key actors (second set of tools) were tape-recorded and transcribed for analysis. The preliminary report of findings was presented during a regional workshop, to all national malaria authorities and other regional technical advisors from different international cooperation agencies. Countries that felt they should score higher in a specific criterium were given the opportunity to submit means of verification to support their claim (i.e. reports of specific studies or evaluation, copy of guidelines for quality control of labs). Final decisions on scores relied entirely within the principal research team.

## Results

### Residual spraying

All the countries studied reported incomplete information relative to the aspects of research, coverage and quality of the residual spraying programme.. Through interviews of key employees in each country, it was determined that the countries had not in general carried out a systematic implementation according to the technical criteria established by the WHO. Table 5 presents satisfaction of the criteria for each of the countries studied. Note that Guyana reported it does not have a residual spraying programme. As per the adequacy scale, one country scored as adequate implementation, another intermediate implementation and the rest scored deficient implementation. The adequacy scale grade is consistent with the perceptions of the technicians and regional officials. For example, a technical adviser who has worked in most of the countries in the region stated:

Vector control is not systematic in any country and it is carried out with very little evidence. In general, houses with the minimum quality for the spraying to work also have access to diagnosis and treatment. The remote population lives with diagnostic and treatment problems, in houses that do not satisfy the conditions for spraying. Spraying continues to be out of reach for the population that needs it most.

**Table 5:** Results of the evaluation of adequacy criteria in the implementation of the residual spraying strategy

No.	Criterion	Ecuador	Peru	Bolivia	Colombia	Guyana
1	Population at risk stratified according to the burden of the disease and epidemiology of the transmission	0	0	0.5	1	N/A

2	Vector habits studied and confirmed	0	0	0.5	1	N/A

3	Sensitivity to potential insecticides confirmed before selecting the insecticide(s) with the best results	0	0	0.5	1	N/A

4	100% of target homes (according to national standards) sprayed at least once a year	0.5	0	0.5	0,5	N/A

5	Stockouts of insecticides for spraying did not exceed six months in any of the cases	1	0	1	1	N/A

6	Updated standards and programmes exist to implement residual spraying	1	0	1	1	N/A

7	A system exists for monitoring resistance and sensitivity to insecticides used in indoor spraying	0	1	1	0.5	N/A

8	Systematic procedures exist for monitoring vector habits	0	1	1	1	N/A

9	Systematic procedures are in place for monitoring residual insecticide in the homes sprayed	0	1	0	0.5	N/A

	Total	2.5	3.0	6.0	7.5	

*Note: *1 = Yes; 0 = No; 0.5 = Partial; N/A = not applicable

Another adviser with experience in all the countries in the region noted the following:

In many of the countries of this region, it is a matter of a lack of entomologists. We have to try to strengthen the entomology in order to make decisions based on evidence of vector control so we are not fumigating without knowing whether the insecticides are working.

A national official from one of the countries recognized the difficulties of implementing the spraying strategy as follows:

Fumigation and spraying are not carried out according the guidelines or target populations. In many cases, it depends on whether the municipalities have requested from authorities fumigation before a local fair or any other celebration. This is done more [often] through political decisions without following the technical criteria of the programme.

### Insecticide-treated bed nets

The five countries have used this strategy relatively recently, with special features in each country. For example, whereas Bolivia and Colombia reported to have carried out studies on stratification of the population, vector habits, and sensitivity to insecticides prior to introducing bed nets, other countries reported to having introduced insecticide-treated bed nets without conducting these studies. Although bed nets were distributed in all the countries, none satisfied the 80% coverage criterion for the countries' target populations. Table 6 presents the scores for each of the countries.

**Table 6:** Results of the evaluation of adequacy criteria in the implementation of the insecticide-treated bed net strategy

No.	Criterion	Ecuador	Peru	Bolivia	Colombia	Guyana
1	Population at risk stratified according to the burden of the disease and epidemiology of the transmission	0	0	1	1	1

2	Vector habits studied and confirmed	0	0	1	1	0

3	Sensitivity to potential insecticides confirmed before selecting the insecticide(s) with the best results	1	0	1	1	0

4	80% of at-risk population received insecticide-treated bed nets	0.5	0.5	0.5	0.5	0.5

5	Insecticide-treated bed nets distributed to 80% of pregnant women in the risk area	0.5	0.5	0.5	0.5	0.5

6	Insecticide-treated bed nets distributed to 80% of children under age five in risk areas	0.5	0.5	0.5	0.5	0.5

7	80% of those surveyed reported sleeping under a bed net the night before	0	0.5	0.5	0	0.5

8	Stockouts of insecticides for impregnation did not exceed three months in the past five years	0	0	N/A	1	0.5

9	Stockouts of new bed nets for delivery to the population did not exceed six months in any of the cases	0	0	0	1	0

10	Standards and programmes exist for home and community-level reimpregnation	0	0	N/A	1	0

11	A systematic procedure exists to monitor that families have bed nets and use them appropriately (including re-impregnation and washing)	0	0.5	1	1	1

12	A system exists for monitoring resistance and sensitivity to insecticides used in bed nets	0	0	1	0	0.5

13	Systematic procedures exist for monitoring vector habits	0	0	1	1	0.5

14	Systematic procedures are in place for monitoring residual insecticide in bed nets	0	0	0	0	0.5

	Total	2.5	2.5	8.0^a^	9.5	6.0

*Note: *1 = Yes; 0 = No; 0.5 = Partial; N/A = not applicable

a. The total in Bolivia is for 12 criteria because the country does not re-impregnate bed nets (long-lasting insecticide-treated bed nets are used)

On the adequacy scale, two countries achieved intermediate grade, and three obtained a deficient grade. One of the national officials interviewed explains the difficulties of the programme as follows:

Delivery of insecticide-treated bed nets is confirmed, but it does not follow technical criteria. Political officials deliver them during visits to settlements, without noting that they are not target areas for bed net delivery.

### Timely diagnosis

The timely diagnosis strategy includes microscopic diagnosis and rapid diagnostic tests, except in the case of Guyana, which has not introduced rapid tests, and where the analysis refers only to microscopic diagnosis. In general, microscopic diagnosis has been the primary strategy in the countries and has increased over the years. All the countries have national guidelines for it and systems to monitor the quality of microscopic diagnosis.

The rapid test strategy is relatively new. Even though it has been introduced and distributed in all Amazonian countries (except Guyana), the introduction and use have not been systematic. Some countries have national standards for the purchase and use of rapid tests, and in countries where they exist, systematic processes for monitoring compliance with shipment, storage and use standards have not been fully implemented [15]. Table 7 presents the compliance with each of the criterion in the countries. As per the adequacy scale, only one country scored as adequate and all the rest scored as deficient.

**Table 7:** Results of the evaluation of adequacy criteria in the implementation of the timely diagnosis strategy

No.	Criterion	Ecuador	Peru	Bolivia	Colombia	Guyana
1	Minimum of 80% of all cases diagnosed in first 24 hours (time between taking the thick film or rapid test and delivery of results in endemic areas)	1	0.5	0.5	0.5	0.5

2	No stockouts of rapid tests in any public network facilities in endemic areas	0	0	0	1	N/A

3	A system exists for monitoring the quality of microscopic diagnosis in the public network	1	1	1	1	1

4	A system exists for monitoring the quality of rapid tests	0	0	0	1	N/A

5	National standards exist for use, distribution, shipment and storage of rapid tests	1	0	0	1	N/A

6	A process exists for monitoring compliance with distribution, shipment and storage standards	0	0	0	0.5	N/A

7	There is some type of training and supervision programme for the staff that uses rapid diagnostic tests	0	0	0	1	N/A

	Total	3.0	1.5	1.0	6.0	1.5^a^

*Note: *1 = Yes; 0 = No; 0.5 = Partial; N/A = not applicable

a. The Guyana evaluation is on two criteria only. Rapid tests are not used in the country

### Artemisinin-based combination therapy

The ACT strategy stands out because of its systematic implementation in all the countries and the collaboration among all of them to ensure preliminary research and studies, coverage and quality for it. The evidence gathered identifies systematic implementation, which is the result of the support that the Amazon Malaria Initiative-Amazon Network for the Surveillance of Antimalarial Drug Resistance has provided to all the countries in the region. This result is documented in publications, reports on resistance and sensitivity studies, and national guidelines [16] It was also acknowledged by public officials interviewed in each of the countries. One of the national authorities expressed:

In ACTs, we have gone step by step with technical fundaments. We started with the studies on antimalarial resistance for the previous treatment scheme. Those studies demonstrated serious problems with resistance. With that info, we revised national treatment schemes and updated national guidelines and training of personnel.

In terms of adequacy criteria, all the countries satisfied the majority of the desired criteria. The criteria not yet satisfied, and common to all the countries, correspond to ensuring the continuous supply of ACT and the implementation of systematic processes to monitor the appropriate application of standards and protocols. As a result of implementation satisfying the majority of the criteria, three countries achieved the grade of 'adequate implementation' and the remaining two 'intermediate implementation' of the strategy, according to the technical criteria established by the WHO. Table 8 presents the grades of the countries for each of the criteria.

**Table 8:** Results of the evaluation of adequacy criteria in the implementation of the ACT strategy

No.	Criterion	Ecuador	Peru	Bolivia	Colombia	Guyana
1	*In vivo *or *in vitro *studies on the resistance of *P. falciparum *to medications in previous treatment plans carried out	1	1	1	1	1

2	*In vivo *or *in vitro *studies on the sensitivity of *P. falciparum *to ACT were carried out	1	1	1	1	1

3	Minimum of 80% of *P. falciparum *cases receive ACT	1	1	1	1	1

4	No ACT stockouts in the public network in the last three years	0	0	0	0.5	0

5	Updated standards and protocols for ACT in treatment plans in the country	1	1	1	1	1

6	There is a system for monitoring ACT treatment failures	1	1	1	1	1

7	According to standards, ACT is only given when there is a positive result (microscopic or rapid test) for *P. falciparum*	1	1	1	0.5	1

8	A systematic procedure exists for monitoring the appropriate application of ACT standards and protocols	1	0.5	0.5	0	0

	Total	7.0	6.5	6.5	6.0	6.0

*Note: *1 = Yes; 0 = No; 0.5 = Partial

According to the data collected, the ACT supply issues are associated with the complexity of implementing procurement processes for new medicines that meet national procurement standards and the progressively smaller volume required for these medicines. For example, a reduced number of vendors has caused the government procurement of ACT to be declared void on several occasions in Peru. Procurement processes involving Ministry of Health units different from the malaria programme have lengthened the processes in the other countries. These problems cause ACT stockouts in the countries not resulting directly from a lack of action on the part of persons responsible for malaria control in the countries, but rather owing to the existing government contracting and procurement processes. A technical advisor who has worked in several countries in the region explains it as follows:

One of the biggest weaknesses at this time is the management of medicines and supplies. Peru and Ecuador have had major stockouts. These problems correspond to the countries' laws on procurement and purchasing, which supersede the malaria programmes. Therefore, in order to make improvements in this area, we must work on the country level, and with central officials from the Treasury and others.

In addition to the preceding issues, decentralization and other health reforms have created a new organizational architecture in these countries, which also adds challenges to the planning, procurement, distribution, and management of supplies. While national malaria control programmes used to be exclusively responsible for the procurement of supplies, this now involves several other units in the Ministries of Health and in some countries, even the Ministry of Finance for international and national tender processes [16].

## Conclusions

The evaluation of the adequacy level of the implementation of the four strategies to control malaria revealed that indoor residual spraying and insecticide-treated bed nets are implemented on a level between intermediate and deficient. The timely diagnosis strategy was also evaluated as being deficient in all the countries except one, where the level was adequate. The ACT strategy presented better scores in all countries because almost all the technical criteria for implementing the strategy have been met. The criteria not yet satisfied are those related to systems to ensure a continuous supply of ACT and systems to oversee quality in the implementation of ACT, including adherence to treatment. In summary, whereas the ACT control strategy has been introduced and implemented following a systematic approach and technical guidelines, the other three of the malaria control strategies present major gaps in implementation according to the technical, coverage and quality criteria used in this study.

Even though the incidence of malaria in the region has shown a significant decline in the past few years, there is no way to ensure that this trend will continue and that there is no risk of starting a cycle of increase in the incidence, as occurred in earlier decades. Therefore, the findings of this study are relevant when one considers that to have lasting control of malaria, the combined impact of the different strategies [17] and of the adequate implementation of each one is necessary. In addition, the current landscape of regions with high and low transmission, which the countries display, requires all the tools and technologies available, as well as adjustment of the strategies and activities to this epidemiological context. The importance of an adequate implementation of the control strategies to maintain the current achievements, avoid outbreaks, and possibly move toward the elimination of malaria in the region cannot be exaggerated.

The results of this study will help countries plan and implement activities to improve the performance of malaria control strategies. One element that must be taken into account is that the paradigm of vertical health programmes, or one national health authority with complete control of the malaria programmes, no longer exists in the region. In the last 15 years, most of the countries in the region have been implementing processes to reform their health systems, which had initially compromised, to varying degrees, the performance of the malaria control programmes [18,19]. Although reforms, particularly descentralization, have imposed enourmous challenges to control programmes, they should not considered to be only negative. Some of the reforms are seeking to integrate control services with general health care delivery at district level facilities; others to have unified procurement of drugs and medical supplies to improve efficiency [20]. These types of reforms are important because there is nowadays, a clear understanding that sustainable control and elimination of malaria cannot occur in isolation from existing health systems. Moreover, health systems strengthening are a key intervention to support the global malaria eradication agenda [21]. The strengthening of control strategies and programmes must consider the new landscape of health systems in the region.

Finally, it is important to be clear about the limiting factors of the specific framework developed for this study. The criteria selected for each intervention and the grading scale were agreed upon by the researchers. Other researchers may select different criteria and a different grading scale. This has implications for the standardization of the tools that are part of our framework. However, this limiting factor is reduced if one considers that the purpose of our approachs is to provide tools to the national control programmes that enable them to identify their own level of performance. Further adaptations to the context of other regions and countries are, therefore, encouraged. The evaluation of malaria control strategies using adequacy criteria is a methodology that is simple, easy to apply, and a useful tool as a preliminary step to implementing more complex and expensive evaluations. In this regards, the specific framework developed is in line with the recent call for an improved research agenda for malaria eradication that includes the development and validation of tool kits and rapid assessment procedures allowing the analysis of bottlenecks for effective coverage of malaria interventions and the degree of integration of interventions into existing health systems [21].

## Authors' contributions

WF wrote the initial manuscript; JC and EB made substantial contributions and helped finalize the manuscript. All authors read and approved the final manuscript.
